# Efficacy and safety of aripiprazole in the treatment of bipolar disorder: a systematic review

**DOI:** 10.1186/1744-859X-8-16

**Published:** 2009-07-27

**Authors:** Konstantinos N Fountoulakis, Eduard Vieta

**Affiliations:** 1 Third Department of Psychiatry, School of Medicine, Aristotle University of Thessaloniki, Thessaloniki, Greece; 2 Bipolar Disorders Program, Hospital Clinic, University of Barcelona, IDIBAPS, CIBERSAM, Barcelona, Spain

## Abstract

**Background:**

The current article is a systematic review concerning the efficacy and safety of aripiprazole in the treatment of bipolar disorder.

**Methods:**

A systematic Medline and repositories search concerning the usefulness of aripiprazole in bipolar disorder was performed, with the combination of the words 'aripiprazole' and 'bipolar'.

**Results:**

The search returned 184 articles and was last updated on 15 April 2009. An additional search included repositories of clinical trials and previous systematic reviews specifically in order to trace unpublished trials. There were seven placebo-controlled randomised controlled trials (RCTs), six with comparator studies and one with add-on studies. They assessed the usefulness of aripiprazole in acute mania, acute bipolar depression and during the maintenance phase in comparison to placebo, lithium or haloperidol.

**Conclusion:**

Aripiprazole appears effective for the treatment and prophylaxis against mania. The data on bipolar depression are so far negative, however there is a need for further study at lower dosages. The most frequent adverse effects are extrapyramidal signs and symptoms, especially akathisia, without any significant weight gain, hyperprolactinaemia or laboratory test changes.

## Background

The treatment of bipolar illness started with lithium and Frederik Lange in the late 19th century [1]; later John Cade in 1949 [2-4] and Mogens Schou with Poul Christian Baastrup in the 1970s established its effectiveness [5-10]. Its long-term effects are still a matter of debate [11]. Anticonvulsants are also considered to be useful in the treatment of bipolar illness. In spite of what many clinicians believe, there is no class effect for this group in bipolar disorder, since only valproate carbamazepine and lamotrigine have strong data support. The use and usefulness of antidepressant agents in bipolar disorder (BD) is controversial. Guidelines suggest their cautious use and always in combination with an antimanic agent [12]. This is because antidepressants are believed to induce switching to mania or hypomania [13-16], mixed episodes [17] and rapid cycling, while research suggests that the use of antimanic agents might protect from such an effect at least partially [18].

The most recent advances in bipolar treatment concern the European Medicines Agency (EMEA) and the US Food and Drug Administration (FDA) approval of olanzapine, risperidone, quetiapine, ziprasidone and aripiprazole for the treatment of acute mania, the approval of quetiapine and the olanzapine-fluoxetine combination against acute bipolar depression and the approval of olanzapine, quetiapine and aripiprazole for the maintenance phase. This is an important development, because the treatment of BD is as difficult and complex as the illness itself [12,19-23]. The low reliability and validity of psychiatric diagnosis perplexes the problem and makes the gathering of scientific data difficult, because the diagnosis of BD in particular is often made retrospectively and carries the risk of memory distortions and biases. It is also well established that an additional problem is that a specific and different treatment needs to be considered separately for manic, hypomanic, mixed and bipolar depression episodes, as well as for unipolar depression [19,21,24]. The complexity of treatment approaches and the problems with data are reflected in treatment reviews [25] and treatment guidelines [12].

First generation (typical) antipsychotics (FGAs) were used since the 50 s, especially for the treatment of acute mania. However the anecdotal clinical impression many psychiatrists have is that they induce depression. This was recently supported by two studies [26,27].

Atypical second generation antipsychotics (SGAs) appear to have broadly similar efficacy as FGAs against the manic symptoms of bipolar disorder, but there are important differences in their tolerability profiles, which are likely to be of particular relevance during long-term treatment [24]. Almost all have received regulatory approval for use in mania, including aripiprazole [28]. If the patient has a life history of predominant manic or mixed episodes with rare and short depressive episodes, the administration of an SGA alone could be enough to control the disorder [29]. Some SGAs seem to also be effective against acute bipolar depression and SGAs in general do not seem to induce switching towards the depressive pole. The biggest problem with some SGAs is weight gain, hyperlipidaemia and diabetes in a significant percentage of the patients receiving them. Various strategies have been developed to cope with this problem, with varying results so far.

The current article attempts to summarise the data available on the usefulness of aripiprazole, which is virtually a third generation antipsychotic (TGA), and a partial dopamine agonist.

## Methods

A systematic Medline and repositories search of clinical trials.gov, Cochran collaboration and industry web sites concerning the usefulness of aripiprazole in bipolar disorder was performed, with the combination of the words 'aripiprazole' and 'bipolar'. A selective review was also performed to locate papers with information directly or indirectly related to the use of aripiprazole in bipolar disorder.

## Results

The search returned 184 articles for randomised controlled trials (RCTs) and was last updated in 15 April 2009. An additional search included repositories of clinical trials and previous systematic reviews in order to trace unpublished trials in particular. There were four RCTs comparing aripiprazole to placebo in acute mania (one unpublished and one without results), three placebo-controlled comparisons to lithium (one) and haloperidol (two) in acute mania, one RCT of intramuscular aripiprazole in acute manic agitation, two placebo-controlled RCTs against bipolar depression, one placebo-controlled RCT and two placebo-controlled RCTs comparing aripiprazole to haloperidol and lithium in maintenance and one placebo-controlled adjunctive aripiprazole to lithium or valproate against acute mania (Table 1).

**Table 1:** Randomised controlled trials (RCTs) assessing the usefulness of aripiprazole in bipolar disorder

							**Patients (n)**			
**Trial no.**	**Publication**	**Target**	**Duration**	**Comparator**	**Placebo**	**Add on**	**Agent**	**Comparator**	**Placebo**	**Comments**

CN138-009	Keck *et al.*, 2003 [68]	Mania	3 weeks	No	Yes	No	130		132	Agent > placebo

CN138-074/NCT00036101	Sachs *et al.*, 2006 [69]	Mania	3 weeks	No	Yes	No	137		135	Agent > placebo

CN138-135/NCT00095511	Keck *et al.*, 2009 [70]	Mania	12 weeks	Lithium	Yes	No	155	160	165	Agent = comparator > placebo

CN138-162/NCT00097266	Young *et al.*, 2009 [71]	Mania	12 weeks	Haloperidol	Yes	No	167	165	153	Agent = comparator > placebo

CN138-007	unpublished	Mania	3 weeks	No	Yes	No	267		134	Agent = placebo

CN138-013	Zimbroff *et al.*, 2007 [74]	Mania	24 h	Lorazepam	Yes	No	156	70	75	Agent = comparator > placebo

CN138-077/NCT00046384	Unpublished	Mania	3 weeks	No	Yes	No	29		27	No results

CN138008	Vieta *et al.*, 2005 [72]	Mania	12 weeks	Haloperidol	No	No	175	172		Agent > comparator

CN138-134/NCT00257972	Vieta *et al.*, 2008 [73]	Mania	6 weeks	No	Yes	Lithium/Vpx	253		131	Agent > placebo

CN138-096/NCT00080314	Thase *et al.*, 2008 [75]	Bipolar depression	8 weeks	No	Yes	No	186		188	Agent = placebo

CN138-146/NCT00094432	Thase *et al.*, 2008 [75]	Bipolar depression	8 weeks	No	Yes	No	187		188	Agent = placebo

CN138-010/NCT00036348	Keck *et al.*, 2006/7 [76,77]; Muzina *et al.*, 2008 [78]	Maintenance	26 to 100 weeks	No	Yes	No	77		83	Agent > placebo

CN138008	Unpublished	Maintenance	14 weeks	Haloperidol	No	No	30	9		No results

CN138-135/NCT00095511	Unpublished	Maintenance	40 weeks	Lithium	No	No	56	38		Agent = comparator

Vpx: Valproex

### Basic facts about aripiprazole

Aripiprazole (7-(4-[4-(2,3-dichlorophenyl)-1-piperazinyl]butyloxy)-3,4-dihydro-2 (1H)-quinolinone (OPC-14597), is a derivative of the dopamine autoreceptor agonist 7-(3-[4-(2,3-dimethylphenyl)piperazinyl]propoxy)-2(1H)-quinolinone (OPC-4392) [30,31], and was developed by Otsuka Ltd in Tokyo, Japan and was first approved by the US FDA in 2002 for the treatment of schizophrenia. Recently it received approval for the treatment of acute manic and mixed in patients with bipolar I disorder and for the maintenance treatment of bipolar patients with a recent manic or mixed episode who had been stabilised and then maintained for at least 6 weeks. It is also approved as an adjunct agent for the treatment of depression.

The mechanism of action of aripiprazole differs both from FGAs as well as from SGAs, and it seems to be a dopamine D2 partial and selective agonist [32]. This gives aripiprazole the privilege to be considered as the first of a new group of antipsychotic agents, the TGAs (or dopamine stabilisers or dopamine partial agonists) [33,34].

More specifically, aripiprazole seems to exhibit typical antagonism at D2 receptors in the mesolimbic pathway, as well as having unique partial agonist activity at D2 receptors in the mesocortical pathway [35]. Whether it is an agonist or antagonist depends mainly on the environment [36,37]. The occupancy at D2 and D3 receptors is high (from 71% at 2 mg/day to 96% at 40 mg/day) [38-41]. However although there seems to be a high occupancy of D3 receptors [42] with some observable effects at least *in vitro *[38], suggesting an indirect effect on D2 via D3 [43], their role concerning the clinical effects of aripiprazole is disputed [44,45]. Short-term clinical trials reported a very low incidence of extrapyramidal symptoms, with akathisia being the most common [46]. Bridging the above, imaging studies reported that aripiprazole occupies up to 95% of striatal D2-like dopamine receptors at clinical doses, and at the same time the incidence of extrapyramidal side effects is no higher than with placebo [39,47]. Because of this special mechanism of function, aripiprazole does not cause upregulation of D2 receptors or an increase in expression of the c-fos mRNA in the striatum, thus having a low risk for extrapyramidal side effects (EPS) [48].

There was some concern that presynaptic dopamine autoreceptor agonists (in spite of being efficacious in the treatment of psychosis) might potentially increase the risk for exacerbation of psychosis through stimulation of postsynaptic dopaminergic receptors. However, the unique issue with aripiprazole is that it acts as a presynaptic D2 agonist and simultaneously has an antagonistic effect at the postsynaptic D2 receptors [48]. It is very interesting that there is evidence that aripiprazole increases dopamine activity in the frontal cortex [49]. While most SGAs bind preferentially to extrastriatal receptors, aripiprazole has high binding rates throughout the brain.

Aripiprazole is also a partial agonist at the 5-hydroxtryptamine (5-HT)1A receptor [50,51], and an antagonist at the 5-HT2A receptor [34,52-54] It has moderate affinity for histamine and α-adrenergic receptors and for the serotonin transporter, and no significant affinity for cholinergic muscarinic receptors [52,55-57]. Recent studies have questioned the role of the 5-HT-mediated systems in the mechanism of action of aripiprazole [33].

Aripiprazole reaches peak concentration (C_max_) 3 to 5 h after ingestion, and has a bioavailability of 87% and half life of 75 h. [58]. It exhibits linear pharmacokinetics and it is administered once daily [59]. Steady-state plasma concentrations are achieved after 2 weeks.

It undergoes extensive hepatic metabolisation (dehydrogenation, hydroxylation, and *N*-dealkylation), mainly by cytochrome P450 2D6 (CYP2D6) and cytochrome P450 3A4 (CYP3A4) [60]. The parenteral drug is excreted only in traces. There is only a minor effect of hepatic or renal failure on the pharmacokinetics of aripiprazole and no adjustment of dosage is recommended [61]. There is only one active metabolite (dehydroaripiprazole) [62], which typically accumulates to approximately 40% of the aripiprazole concentration [61,63]. Its elimination half life is about 94 h. All the metabolites, either active or not, are excreted via faeces and urine.

A multicentre, open-label, sequential-cohort, dose-escalation study that explored the tolerability and pharmacokinetics of aripiprazole up to 30 mg/day in 21 children and adolescents (aged 10 to 17 years), preferentially with primary psychiatric diagnoses of a bipolar or schizophrenia spectrum disorder, reported that aripiprazole treatment was generally well tolerated with criteria for tolerability met for all doses tested. There were no serious side effects, deaths or clinically relevant changes in vital signs or weight, and aripiprazole pharmacokinetics seemed to be linear across the tested dose range [64].

Since aripiprazole is metabolised by CYP2D6 and CYP3A4, the coadministration with medications that inhibit (for example, paroxetine, fluoxetine) or induce (for example, carbamazepine) these metabolic enzymes alters its plasma levels. Concomitant alcohol use could lead to an increase of the sedative effects and decrease of the euphoric effects of alcohol [65], but the latter has not been replicated [66].

The common side effects during aripiprazole treatment include akathisia, tremor, headache, dizziness, somnolence, sedation fatigue, nausea, vomiting, dyspepsia, constipation, light-headedness, insomnia, restlessness, sleepiness, anxiety, hypersalivation and blurred vision. The uncommon side effects, and those whose frequency is not precisely known, include uncontrollable twitching or jerking movements, seizures, weight gain, orthostatic hypotension or tachycardia, allergic reaction (such as swelling in the mouth or throat, itching, rash), speech disorder, agitation, fainting, transaminasaemia, pancreatitis, muscle pain, stiffness, or cramps and very rarely neuroleptic malignant syndrome and tardive dyskinaesia. Elderly patients with psychosis associated with Alzheimer's disease and treated with aripiprazole are at increased risk of death compared to placebo due to cardiovascular (for example, heart failure, sudden death) or infectious (for example, pneumonia) problems or stroke.

It is interesting that the investigation of the effect of aripiprazole (2.0 to 8.0 mg/kg, orally) vs olanzapine (1.0 to 10 mg/kg, orally) on bodyweight in an animal model in two strains (Wistar and Sprague-Dawley) and under two housing conditions (single and group housed) revealed that subchronic aripiprazole treatment resulted in rapid and robust weight gain similar to those observed with olanzapine. In spite of similar effects on bodyweight, aripiprazole and olanzapine stimulated markedly different patterns of prolactin secretion. Bodyweight changes and prolactin secretion induced by these antipsychotics were significantly modulated by housing and by strain [67].

### Aripiprazole in bipolar disorder

#### RCTs

##### Acute mania

Aripiprazole was studied against acute mania in five RCT studies involving a placebo arm. In the first (CN138-009), which was published in 2003 [68], 262 patients with a manic or mixed episode, randomised 1:1 to aripiprazole or placebo, took part. The study duration was 3 weeks and the mean aripiprazole dosage utilised was 27.9 mg daily, with 86% of patients receiving the maximum dosage of 30 mg daily. The use of lorazepam was similar in both groups. Overall, 82 (31%) completed the 3-week trial duration and the completion rate was higher for aripiprazole in comparison to placebo (42% vs 21%, *P *< 0.001). Aripiprazole significantly reduced the Young Mania Rating Scale (YMRS) score by more in comparison to placebo (-8.2 vs -3.4, *P *= 0.002) and the Clinical Global Impression (CGI) score for mania (-1.0 vs -0.4, *P *= 0.001), depression (-0.2 vs +0.1.4, *P *= 0.03), and overall bipolar illness (-1.0 vs -0.4, *P *= 0.001). These data are indirectly suggestive of an effect on mixed episodes as well. The therapeutic effect was evident from day 4. The response rate was significantly higher in the aripiprazole group in comparison to placebo at endpoint (40% vs 19%). Headache was the most frequent adverse event, but levels were similar in both groups (36% vs 31%). The following events were more frequent in the aripiprazole group: nausea (23% vs 10%), dyspepsia (22% vs 10%), somnolence (20% vs 5%), anxiety (18% vs 10%), vomiting (16% vs 5%), insomnia (15% vs 9%), light-headedness (14% vs 8%), constipation (13% vs 6%), accidental injury (12% vs 2%) and akathisia (11% vs 2%). Two patients with aripiprazole had an increase in bodyweight of more than 7% versus none in the placebo group. Only 11% in the aripiprazole group had high prolactin levels at endpoint in comparison to 17% in the placebo group, while at baseline the respective values were 30% and 23%.

The second placebo-controlled RCT (CN138-074/NCT00036101) [69] was published in 2006 and it included 272 patients with a manic or mixed episode, randomised 1:1 to aripiprazole or placebo and lasted for 3 weeks. The mean aripiprazole dosage utilised was 27.7 mg daily, with 85% of patients receiving the maximum dosage of 30 mg daily. The use of lorazepam was again similar in both groups. Overall, 145 (53%) completed the 3-week trial duration and the completion rate was similar for both study groups (55% vs 52%). Aripiprazole significantly reduced the YMRS score by more in comparison to placebo (-12.5 vs -7.2, *P *< 0.001) and the CGI score for mania (-1.59 vs -1.12, *P *< 0.01), depression (-0.60 vs -0.31, *P *< 0.05), and overall bipolar illness (-1.42 vs -0.97, *P *< 0.01). The separate analysis of patients with rapid cycling suggested that aripiprazole significantly reduced the YMRS score in this group (-15.27 vs -5.45, *P *= 0.002) but not the Montgomery Åsberg Depression Rating Scale (MADRS) score. The same analysis concerning the patients with mixed episodes revealed a significant effect on both the YMRS score (-14.12 vs -9.02, *P *= 0.01) and the MADRS score (-7.93 vs -4.29, *P *= 0.041). All therapeutic effects on the total sample and the subgroups of rapid cycling and mixed episodes patients were all evident from day 4. The response rate was significantly higher in the aripiprazole group in comparison to placebo at endpoint (53% vs 32%). Headache was the most frequent adverse event, but similar in both groups (25% vs 24.8%). Similar rates were also registered for anxiety (10.3% vs 8.3%) and light-headedness (8.8% vs 10.5%). The following events were more frequent in the aripiprazole group: nausea (21.3% vs 15.8%), dyspepsia (15.4% vs 6.8%), somnolence (19.9% vs 10.5%), vomiting (11% vs 7.5%), constipation (11.8% vs 5.3%) and akathisia (17.6% vs 4.5%). One patient with aripiprazole had an increase in bodyweight of more than 7% versus five in the placebo group. Only 4% in the aripiprazole group had high prolactin levels at endpoint in comparison to 11% in the placebo group.

The third placebo-controlled RCT (CN138-135/NCT00095511) [70] was published in 2008 and compared aripiprazole to lithium and placebo for 3 weeks and aripiprazole and lithium for an additional 9 weeks. It included 480 patients with a manic or mixed episode, randomised 1:1:1 to aripiprazole lithium or placebo for 3 weeks and those on placebo were afterwards randomised 1:1 to either lithium or aripiprazole. Rapid cycling patients were excluded from the study. The mean aripiprazole dosage was 23.2 mg daily at the end of week 3 and 23.6 mg daily at week 12. The respective dosages for lithium were 1,146.9 mg and 1,210.6 mg daily, corresponding to serum levels of 0.76 mEq/litre and 0.66 mEq/litre, respectively. The use of anxiolytics was similar in all groups (87.2% to 91.6%). Overall, 229 (47.7%) completed the 3-week trial duration and the completion rate was similar for all study groups (placebo 47%, aripiprazole 47%, lithium 49%). At week 12 there were 143 patients (30%) still in the study (placebo 28.5%, aripiprazole 27%, lithium 33.7%). Aripiprazole and lithium significantly reduced the YMRS score by more in comparison to placebo at week 3 (-12.6 and -12.0, respectively vs -9.0 for placebo, *P *< 0.001) and this effect was maintained at week 12 (-14.5 and -12.7, respectively). There was a significant change in the CGI score for mania (placebo 3.1 vs lithium 2.9 vs aripiprazole 2.5, *P *< 0.01 between placebo and aripiprazole). There were no differences in the MADRS change among groups, while there were some differences in the Positive and Negative Syndrome Scale (PANSS) subscores between aripiprazole and placebo (total PANSS, cognitive and hostility subscales). The therapeutic effect was evident from day 2. On the basis of the YMRS change, the effect size Cohen d was equal to 0.30. The response rate was significantly higher in the aripiprazole and lithium group in comparison to placebo at week 3 (46.8% vs 45.8% vs 34.4%, respectively) and at week 12 there was a slight superiority for aripiprazole in comparison to lithium (56.5% vs 49%, respectively). The remission rates followed a similar pattern both at week 3 (40.3% vs 40.0% vs 28.2%, respectively) and at week 12 there was again superiority for aripiprazole in comparison to lithium (49.4% vs 39.4%, respectively). Headache was the most frequent adverse event, but similar in all groups (placebo 22.6%, lithium 20.1%, aripiprazole 23.4%). The following events were more frequent in the aripiprazole group: nausea (13.4% vs 23.3% vs 22.7%), constipation (6.1% vs 10.73% vs 10.4%), sedation (4.9% vs 6.9% vs 11.7%), tremor (4.9% vs 10.1% vs 7.1%), and akathisia (3.0% vs 5.0% vs 11.0%). One patient with aripiprazole, one on placebo and two on lithium had an increase in bodyweight of more than 7%. Only 8.2% in the aripiprazole group had high prolactin levels at week 12 in comparison to 18.2% in the lithium group.

The fourth placebo-controlled RCT (CN138-162/NCT00097266) [71] was published in 2009 and compared aripiprazole to haloperidol and placebo for 3 weeks and aripiprazole and lithium for an additional 9 weeks. It included 485 patients with a manic or mixed episode, randomised 1:1:1 to aripiprazole haloperidol or placebo for 3 weeks and those on placebo were afterwards put blindly on aripiprazole. Rapid cycling patients were excluded from the study. The mean aripiprazole dosage was 23.6 mg daily at the end of week 3 and 22 mg daily at week 12. The respective daily dosages for haloperidol were 8.5 and 7.4 mg daily. At the end of week 3, 53% of aripiprazole treated patients were receiving 30 mg daily and at week 12 the percentage was 48%. The use of concomitant psychoactive medication was similar in all groups (66% to 77%). Overall, 356 (73.4%) completed the 3-week trial duration and the completion rate was similar for all study groups (placebo 71%, aripiprazole 75%, haloperidol 73%). At week 12 there were 274 patients (56.5%) still in the study (placebo 55%, aripiprazole 57%, haloperidol (58%). Aripiprazole and haloperidol significantly reduced the YMRS score by more in comparison to placebo at week 3 (-11.9 and -12.8, respectively vs -8.7 for placebo, *P *< 0.05) and this effect was maintained at week 12 (-17.2 and -17.8, respectively). There was a significant change in the CGI score for mania (placebo -1.1 vs haloperidol -1.5 vs aripiprazole -1.4, *P *< 0.05). There were no differences in the MADRS change among groups, while there were some differences in the PANSS subscores between aripiprazole and placebo (total PANSS, positive, cognitive and hostility subscales). The therapeutic effect was evident from day 2. The response rate was significantly higher in the aripiprazole and haloperidol group in comparison to placebo at 3 weeks (47% vs 49.7% vs 38.2%, respectively) and at 12 weeks there was a similar rate of response for aripiprazole and haloperidol (72.3% vs 73.9%, respectively). The remission rates followed a similar pattern both at 3 weeks (44% vs 45.3% vs 36.8%, respectively) and at 12 weeks there was again a similarity of rates between aripiprazole and haloperidol (69.9% vs 71.4%, respectively). The following events were frequent in the aripiprazole group: insomnia (13.9%), EPS (7.8%), and akathisia (11.4%). At week 3, there were three patients on aripiprazole, nine on placebo and four on haloperidol that had an increase in bodyweight of more than 7%. At the same time point, 22.4% on aripiprazole vs 66.2% on haloperidole had high prolactin levels and at week 12 the respective percentages were 12.8% and 60.8%.

A fifth trial, (CN138-007) was negative and was not published [28]. It included 267 patients randomised to fixed doses of aripiprazole (131 patients to 15 mg daily and 136 to 30 mg daily) and 134 to placebo for 3 weeks. Total discontinuation was similar in all groups (57% to 60%) and the reasons for discontinuation were also similar. The changes in the YMRS score were -10.01, -10.8 and -10.12, respectively. The CGI changes were 4.66, 4.7 and 4.68, respectively and the PANSS changes were again similar.

The sixth trial (CN138-077/NCT00046384) did not produce any results due to the small number of patients recruited (29 in the aripiprazole arm and 27 in the placebo arm).

In a 12-week RCT (CN138008) of aripiprazole comparison to haloperidol (in a 1:1 ratio. without a placebo arm), in 347 patients with bipolar I disorder experiencing acute manic or mixed episodes (175 in the aripiprazole group and 172 in the haloperidol group), at week 3, the average daily dosage of aripiprazole was 22.6 mg and of haloperidol was 11.6 mg. At week 12, average daily dosages were 21.6 mg for aripiprazole and 11.1 mg for haloperidol. No psychotropic medications including anticholinergics were permitted, except for benzodiazepines during the first few days. Overall, 134 patients receiving aripiprazole and 95 receiving haloperidol completed the first 3 weeks of treatment; the week 12 numbers were 89 and 50, respectively. The response rate was 49.7% in the aripiprazole group and 28.4% in the haloperidol group (*P *< 0.001). The proportion of patients in remission at week 12 was significantly higher in the aripiprazole group than in the haloperidol group (50% v. 27%; *P *= 0.001). The efficacy measures showed similar changes in the aripiprazole and haloperidol groups with both last observation carried forward and observed cases analyses. Significantly more patients demonstrated a 50% or greater decrease in MADRS total score from baseline with aripiprazole than with haloperidol at week 12 (51% v. 33%; *P *= 0.001) and aripiprazole treatment produced greater numerical reductions in depressive symptoms compared with haloperidol, as measured by the mean change in MADRS total score at endpoint. Of 173 patients treated with aripiprazole, 19 (11.0%) switched to depression; of 164 on haloperidol, 29 (17.7%) switched to depression. The most frequent adverse events leading to discontinuation were extrapyramidal symptoms (haloperidol 18.9% vs aripiprazole, 2.9%), and akathisia (haloperidol 14.2% vs aripiprazole 5.1%). During the study, time to discontinuation for any reason was significantly greater for patients receiving aripiprazole than those receiving haloperidol (*P *= 0.001). The most common reason for discontinuation was experiencing adverse events which showed a marked difference in incidence between the groups (aripiprazole 9.7% vs haloperidol 30.8%). The incidence of extrapyramidal adverse events in the haloperidol group (62.7%) was more than double that in the aripiprazole group (24.0%), while the mean change in weight from baseline at endpoint was not significantly different between groups. Serum prolactin levels showed a mean decrease from baseline in the aripiprazole group (-13.4 ng/ml, -284.1 mU/litre), and a mean increase in the haloperidol group (7.7 ng/ml, 163.2 mU/litre) at week 12 (*P *= 0.001). In the haloperidol group, 57.1% of patients experienced serum prolactin levels above the upper limit of normal compared with 14.1% in the aripiprazole group. No clinically meaningful difference was detected in vital sign measurements, laboratory abnormalities or cholesterol levels between the aripiprazole and haloperidol treatment groups [72].

There was a final 6-week placebo-controlled RCT (CN138-134/NCT00257972) with aripiprazole as adjunctive treatment to lithium or valproate [73]. It included 131 patients in the placebo arm and 253 in the aripiprazole arm; 157 were receiving lithium and 227 were receiving valproate. Double-blind treatment was completed by 85% and 79% of patients randomly assigned to placebo and aripiprazole, respectively. Discontinuation rates due to adverse events were higher for patients in the aripiprazole group than for patients in the placebo group (9% vs 5%). The mean dose of aripiprazole during week 6 was 19.0 mg/day. The dosages of lithium and valproate treatment were similar in the placebo and the aripiprazole groups (lithium 994 mg/day and 1,119 mg/day, serum levels 0.77 mmol/litre and 0.78 mmol/litre, respectively; valproate 1,175 mg/day and 1,180 mg/day, respectively). At endpoint, adjunctive aripiprazole showed significantly greater improvements from baseline in YMRS total score than placebo (-13.3 ± 7.9 vs -10.7 ± 7.6, *P *< 0.0) but this was due to the valproate but not the lithium group. At endpoint the remission rate was 66.0% for aripiprazole and 50.8% for placebo (*P *< 0.01, number needed to treat (NNT) = 7). The improvement over placebo in MADRS was not statistically significant at endpoint, however the proportion of patients with emergent depression was significantly lower in the aripiprazole arm than the placebo arm (7.7% vs 16.9%; *P *< 0.01). The most frequently reported adverse event was akathisia (aripiprazole: 18.6% vs placebo: 5.4%), tremor (placebo: 6.2% vs aripiprazole: 9.1%), EPS (placebo: 0.8% vs aripiprazole: 4.7%), hypertonia (placebo: 0% vs aripiprazole: 0.4%), hypokinaesia (placebo: 0% vs aripiprazole: 0.4%), muscle spasms (placebo: 0.8% vs aripiprazole: 2.0%), dyskinaesia (placebo: 0.8% vs aripiprazole: 0.4%) and muscle twitching (placebo: 0% vs aripiprazole: 0.4%). The lithium subgroup showed higher rates of akathisia (aripiprazole: 28.3% vs placebo: 4.0%) and tremor (aripiprazole: 13.2% vs placebo: 8.0%) than the valproate subgroup (aripiprazole: 11.6% vs placebo: 6.3% and aripiprazole: 6.1% vs placebo: 5.0%, respectively). There were no clinically meaningful differences between treatments in weight change and laboratory parameters including serum prolactin levels and electrocardiogram (ECG).

The above studies strongly support the efficacy of aripiprazole against acute mania and they report that the separation from placebo occurred as early as days 2 to 4. They also provide some support for its efficacy against mixed episodes and rapid cycling without switching to depression. Side effects included mainly akathisia, without weight gain, hyperprolactinaemia or corrected QT (QTc) prolongation.

A placebo-controlled RCT was conducted on the efficacy and safety of intramuscular aripiprazole for the treatment of agitation in patients with bipolar I disorder, manic or mixed episodes [74], which included 301 patients of whom 78 were randomised to intramuscular aripiprazole 9.75 mg per injection, 78 to 15 mg per injection, 70 to intramuscular lorazepam 2 mg per injection and 75 to intramuscular placebo. The mean improvements in PANSS-Excited component score at 2 h were significantly greater with aripiprazole (-8.7 for the 9.75 mg group and -8.7 for the 15 mg group) and lorazepam (-9.6) versus placebo (-5.8), with *P *< 0.001. Also for all other efficacy measures, all three active treatments were similar and superior to placebo at 2 h (*P *< 0.05).

### Acute bipolar depression

There are two 8-week placebo-controlled RCTs concerning the use of aripiprazole in acute bipolar depression (non-psychotic bipolar I patients) and they were both negative at study endpoint [75].

The CN138-096/NCT00080314 included 374 patients; 188 on placebo and 186 on aripiprazole (5 to 30 mg daily). More patients in the aripiprazole group (47%) discontinued double-blind treatment than in the placebo group (35%). The mean aripiprazole dose at the end of the study was 17.6 mg daily. The results suggest that in weeks 1 to 6 there was a significant reduction in the MADRS scores in the aripiprazole group in comparison to placebo (-4.55 vs -6.61; -7.55 vs -9.61; -8.88 vs -11.06; -9.7 vs -12.28; -10.54 vs -13.24; -9.98 vs -12.66 for weeks 1 to 6, respectively), but at week 7 this difference disappeared (-11.14 vs -11.86) and eventually the active agent was no better than placebo. Hypnotics and sedatives were used by 25% of aripiprazole patients and 21% of placebo patients. At least 1 adverse event was reported by 142 (76%) of the 186 patients in the placebo group and 154 (87%) of the 178 patients in the aripiprazole group. The most frequent adverse events (≥5% incidence in the aripiprazole group and twice the incidence in the placebo group) were akathisia (28%), insomnia (16%), nausea (15%), fatigue (11%), back pain (8%), dry mouth (8%), increased appetite (7%), vomiting (6%), anxiety (6%), and sedation (5%). One suicide attempt occurred in the placebo group and one patient in the aripiprazole group manifested severe suicidal ideation. In addition, three non-serious suicidal ideation events were reported in the aripiprazole group. EPS-related adverse events occurred in 18 (9.7%) of placebo-treated patients and 61 (34.3%) of aripiprazole-treated patients. Of the EPS-related adverse events, only akathisia was reported at a frequency of ≥10% in the aripiprazole group and at least twice the rate of placebo.

The CN138-146/NCT00094432 included 375 patients; 188 on placebo and 187 on aripiprazole (5 to 30 mg daily). More patients in the aripiprazole group (41.2%) discontinued double-blind treatment than in the placebo group (29.8%). The mean aripiprazole dose at the end of the study was 15.5 mg daily. The results suggest that in weeks 1 to 3 and then again at week 5 there is a significant reduction in the MADRS scores in the aripiprazole group in comparison to placebo (-4.97 vs -6.54; -6.75 vs -9.52; -9.26 vs -11.65 and -10.47 vs -13.33, for weeks 1 to 3 and 5, respectively), but at endpoint this difference disappeared (-11.46 vs -12.34) and eventually the active agent was no better than placebo. Hypnotics and sedatives were used by 24% of aripiprazole patients and 17% of placebo patients. At least 1 adverse event was reported by 132 (72.9%) of the 181 patients in the placebo group and 155 (85.2%) of the 182 patients in the aripiprazole group. The most frequent adverse events (≥5% incidence in the aripiprazole group and at least twice the incidence in the placebo group) were akathisia (21.4%), restlessness (12.1%), anxiety (9.3%), disturbance in attention (5.5%), and sedation (5.5%). One suicide attempt occurred in the placebo group and one severe adverse event of suicidal ideation occurred in the aripiprazole group. EPS occurred in 19 (10.5%) of placebo-treated patients and 54 (29.7%) of aripiprazole-treated patients. Of the EPS-related adverse events, only akathisia was reported at a frequency of ≥10% in the aripiprazole group and at least twice the rate of placebo.

There were no clinically relevant changes in weight gain, serum prolactin from baseline to endpoint and no differences between the two treatment groups in potentially clinically relevant vital sign or ECG abnormalities. The higher discontinuation rates in the aripiprazole group than in the placebo group in both studies demonstrate that the dosing regimen of aripiprazole (5 to 30 mg/day) was not as well tolerated in this patient population as were the dosing regimens used in previous studies in patients with bipolar mania or schizophrenia.

### Maintenance phase

There was one placebo-controlled RCT (CN138-010/NCT00036348) studying aripiprazole in the maintenance phase in bipolar I, recently manic patients [76]. Patients were stabilised with 15 to 30 mg of aripiprazole for 6 to 18 weeks and then randomised to a 1:1 ratio to aripiprazole or placebo for an additional 26 weeks. Only anticholinergics and lorazepam were allowed as concomitant medication. During the 26 weeks 71.1% of patients on placebo and 71.4% of patients on aripiprazole received at least one concomitant medication. The primary efficacy outcome was time to relapse for a mood episode. From a total of 633 patients initially screened and 206 of them who completed the stabilisation phase, 161 were randomised (83 to placebo and 78 to aripiprazole). A total of 39 patients (50%) on aripiprazole and 28 (34%) on placebo completed the 26 weeks of the trial. The mean aripiprazole daily dosage at the end of 26 weeks was 24.3 mg. The time to relapse was significantly longer for aripiprazole (*P *= 0.02) and the hazard ratio (HR) was 0.52. This was due to a prolongation in the time to relapse to a manic (*P *= 0.01, HR = 0.31) but not to a depressive episode. Unfortunately the mean time to relapse for either group was not reported. At the end of the study 49% of the placebo group and 72% of the aripiprazole group had not experienced a relapse to a mood episode. The difference was significant only concerning manic relapses (23% vs 8%; *P *= 0.009). The same was true for the final YMRS score while there was no difference concerning the MADRS score. There was also a superiority of aripiprazole in comparison to placebo concerning the CGI and the PANSS scores.

More patients with a current episode of mania were randomly assigned to placebo (78%) in comparison to aripiprazole (62%), and fewer with mixed episode were randomised to placebo (22%) than to aripiprazole (38%). This means the aripiprazole group had more patients with mixed index episodes, and thus might constitute a more refractory group of patients, especially with respect to the depressive aspect of symptomatology. The adverse events reported by aripiprazole-treated patients at an incidence ≥5% and twice the incidence of placebo during the maintenance phase were tremor (9.1%), akathisia (6.5%), vaginitis (6.4%), and pain in the extremities (5.2%). One aripiprazole-treated patient and one placebo-treated patient attempted suicide in the stabilisation and maintenance phases, respectively. There were no significant differences concerning the QTc, while aripiprazole treated patients showed a significant drop in prolactin levels. Concerning weight gain, 13% of aripiprazole treated patients put >7% of weight in comparison to none in the placebo group.

This same trial (CN138-010/NCT00036348) also included a 74-week placebo-controlled extension phase [77], which included 66 of the 67 patients who completed the 26-week period. Unfortunately only 12 of them (5 in the placebo group and 7 in the aripiprazole group) completed the 74-week treatment period. The reasons for this high discontinuation rate varied and included lack of efficacy, side effects (very low percentage) and most importantly the very design and structure of the study (the study was closed by the sponsor when the prespecified number of relapses had been attained). Because of this and because detailed descriptive data were not reported, arriving at conclusions is very difficult. The mean dosage of aripiprazole at the end of the 74-week period was 23.6 mg daily. It is reported that 29 out of the 66 patients relapsed (16 out of the 39 in the aripiprazole group (41%) and 13 out of the 27 in the placebo group (48.1%)). The only difference concerned manic relapses (nine in the placebo and six in the aripiprazole group). Again the YMRS score significantly differed between groups. The adverse events had a similar rate to the 26-week period.

In both the above reports the median survival time for the aripiprazole group was not evaluable, while the median survival time for placebo was 118 to 203 days depending on the clinical subpopulation [78].

A *post hoc *subgroup analysis [78] of 28 patients (14 on placebo and 14 on aripiprazole) with rapid-cycling bipolar I disorder from the previous maintenance study (CN138-010/NCT00036348) suggested that aripiprazole was more effective than placebo in the prophylaxis of rapid cycling patients against manic/mixed episodes. More specifically, 12 patients (5 on placebo (36%) and 7 on aripiprazole (50%)) completed the 26-week period, and 3 completed the 100-week period (none on placebo (0%), 3 on aripiprazole (21%)). The mean endpoint aripiprazole daily dosage was 25.3 mg for the 26-week phase and 23.6 for the 100-week phase. The time to relapse was significantly longer with aripiprazole vs placebo treatment at both 26 weeks (*P *= 0.033; HR = 0.21) and 100 weeks (*P *= 0.017; HR = 0.18). The median survival time in the placebo group was 118 days at which time approximately 45% of patients had not yet relapsed. The median survival time for the aripiprazole group was not evaluable. At the time of the last relapse event in the study period, which occurred at day 101, 81% of aripiprazole-treated subjects with rapid-cycling bipolar disorder had not yet relapsed. The YMRS total scores increased in both groups and this increase was numerically smaller with aripiprazole vs placebo from at week 26 (+3.0 ± 2.0 vs +6.6 ± 2.0; *P *= 0.213; effect size 0.506) and week 100 (+2.6 ± 2.6 vs +9.5 ± 2.6; *P *= 0.077; effect size 0.730). The same held true for the MADRS total scores, which increased in both treatment groups with no statistically significant difference with aripiprazole versus placebo at week 26 (+8.3 ± 3.3 vs +11.5 ± 3.3; *P *= 0.519; effect size 0.251) or week 100 (+7.7 ± 3.3 vs +12.5 ± 3.3; *P *= 0.304; effect size 0.403).

The extension of the acute phase trial CN138-135/NCT00095511 [70] for an additional 40 weeks (52 weeks in total) comparing aripiprazole to lithium without a placebo arm suggested aripiprazole is equal to lithium in maintenance against manic episodes. Of the original 480 patients during the acute phase, 94 entered the 40-week extension phase (38 from the original lithium group, 25 patients from the aripiprazole group and 31 from the original placebo group who all switched to aripiprazole). The mean daily dose of aripiprazole and lithium during the last 4 weeks of the extension phase were 21.7 mg/day and 1,209.1 mg/day, respectively. A total of 34 of the 94 patients completed the extension phase it; 7 (4.5%) patients in the aripiprazole group, 13 (8.1%) patients in the lithium group, and 14 (8.5%) patients who were randomised to placebo and blindly switched to aripiprazole after week 3. The most frequently occurring treatment-related adverse events in the aripiprazole treatment group were akathisia (8.0%) and headache (8.0%) and in the lithium treatment group was diarrhoea (10.5%). For both treatment groups, the improvement that was observed at the end of 12-week double-blind treatment phase was maintained throughout the extension phase. No clinically relevant trends were reported in laboratory, vital sign, or ECG results during long-term treatment of aripiprazole.

Another trial extension, that for trial CN138008 to a further 14 weeks, failed to provide any results because of a high drop-out rate.

### Other studies and case reports

There are a number of interesting open trials concerning the usefulness of aripiprazole in bipolar depression, especially in refractory cases and also as adjunctive treatment.

A 6-week prospective, non-randomised, open-label study in 20 bipolar depressed outpatients (10 type I, 7 type II, 3 type not otherwise specified (NOS)), with flexible aripiprazole dosage (up to 30 mg daily) reported a response rate of 44% in patients who completed at least 1 week of treatment and a drop-out rate of 35% [79]. The assessment of anhedonia in 50 bipolar I patients revealed it was present in 52% of them and was significantly reduced during treatment with aripiprazole. In this study all patients completed the 16-week trial and 16% of patients experienced side effects (mainly akathisia and headache) [80].

Two open studies examine the response of refractory bipolar depressive patients to aripiprazole monotherapy. In the first, aripiprazole response was prospectively assessed for 8 weeks in 31 patients with acute bipolar depression inadequately responsive to a mood stabiliser. Only 45% completed the 8-week trial and discontinuation was primarily due to side effects. The results suggested that 42% patients responded and 35% remitted [81]. In the second, aripiprazole response was prospectively assessed for 16 weeks in 85 acutely depressed bipolar patients with inadequate responsive to 1 mood stabiliser. In half of them aripiprazole was given as monotherapy. Only 3.5% of patients discontinued the study for side effects while 21.2% of patients experienced akathisia. The trial was completed by 94.1% of patients. The response rate was 65% and the remission rate was 37.5% [82].

An open clinical trial of adjunctive aripiprazole to 30 outpatients with treatment-resistant bipolar depression (11 type I, 15 type II, 4 NOS; mean age 44.4 ± 17.0 years, 70% female) for a mean duration of 84 ± 69 days, and with a mean final dose of 15.3 ± 11.2 led to a 47% discontinuation (17% due to inefficacy, 10% patient choice and 20% due to adverse events) and to 27% response including 13% remission [83]. In an open-label study aripiprazole was coadministered at 10 mg/day for 3 days, 20 mg/day for 3 days, then 30 mg/day for 8 days, with lamotrigine in 18 bipolar patients. The results suggest aripiprazole has no meaningful effect on lamotrigine steady-state pharmacokinetics in patients with bipolar I disorder [84]. An open-label 12-week trial of adjunctive aripiprazole (initiated at 5 mg daily and increased as tolerated) to existing mood stabiliser medication treatment in 20 older adults (aged 50 to 83 years) reported significant improvement in both manic and depressive symptoms with good tolerability [85].

There are two chart review studies on the adjunctive use of aripiprazole in refractory bipolar depressives. The first suggests that the review of 12 patients with treatment-resistant bipolar disorder (I, II and NOS) who received aripiprazole augmentation for the relief of an acute major depressive episode revealed that after 8 weeks of treatment 33% of patients demonstrated a response but 42% of patients newly developed akathisia [86] The results were sustained after 36 months [87]. The second review of chart records of 10 patients with bipolar I depression refractory to mood stabilisers and treated with adjunctive aripiprazole 15 to 30 mg/day for 21 to 110 days suggests that 70% responded [88].

Aripiprazole has also been studied in the treatment of paediatric bipolar disorder. An 8-week, open-label, prospective study of aripiprazole 9.4 ± 4.2 mg/day monotherapy against acute mania in paediatric bipolar disorder in 19 youths reported that 79% completed the study and aripiprazole treatment was associated with significant improvement. There were only two drop out cases due to extrapyramidal symptoms [89]. Another open 6-week trial in 10 children and adolescents with acute mania comorbid with attention-deficit/hyperactivity disorder (ADHD) reported an improvement in global functioning scores and ADHD symptoms. However although in general tolerability was good, significant weight gain was observed [90]. A retrospective medical chart review of 30 child and adolescent bipolar patients with a Diagnostic and Statistical Manual of Mental Disorders, fourth edition (DSM-IV) diagnosis of bipolar type I, type II disorder, NOS, or schizoaffective disorder, bipolar type, and who were treated with aripiprazole (mean starting dose = 9 ± 4 mg/day, mean final dose = 10 ± 3 mg/day) suggested that the overall response rate was 67%. No serious adverse events were identified and the common side effects included sedation (n = 10, 33%), akathisia (n = 7, 23%), and gastrointestinal disturbances (n = 2, 7%). The change in weight ranged from +5 to -21 kg and 86% of patients lost weight [91]. Another retrospective medical chart review of 41 youths (mean age ± standard deviation (SD): 11.4 ± 3.5 years) with bipolar spectrum disorder treated with aripiprazole (mean daily dose of aripiprazole 16.0 ± 7.9 mg over an average of 4.6 months) suggested a response rate of 71% in manic symptoms and the treatment with aripiprazole was well tolerated [92].

Finally, a study on 20 antipsychotic-treated patients with bipolar or schizoaffective disorder and current substance abuse who were switched to open-label aripiprazole over 12 weeks showed an improvement in Hamilton Rating Scale for Depression (HAM-D) (*P *= 0.002), YMRS (*P *= 0.021), Brief Psychiatric Rating Scale (BPRS) (*P *= 0.000) scores as well as a decrease in alcohol and substance craving without a significant change in antipsychotic-induced side effect scales [93].

A number of case reports provide further information concerning the effects and the safety of aripiprazole. There are a limited number of case reports suggesting that tardive dyskinaesia is improved with aripiprazole treatment [94] and this happens also with haloperidol-induced hyperprolactinaemia [95]. In contrast, aripiprazole might lead to neuroleptic malignant syndrome (one case report in coadministration with lithium) [96]. Aripiprazole can induce acute dystonia in younger patients with concomitant drug abuse or Tourette's disorder [97-99], and there is also a case report on a paradoxical motor syndrome following a switch from atypical neuroleptics to aripiprazole [100] There is one case report in the literature concerning the occurrence of severe extrapyramidal symptoms in an adolescent with a history of such symptoms [101] and in a 3-year old child [102]. Specifically, the child weighed 15.5 kg, and adverse events occurred after the accidental ingestion of a single dose of <15 mg of aripiprazole (probably half of a 15 mg pill); the patient was hospitalised 48 h later with extreme lethargy, flat affect, intention tremor, truncal ataxia and Parkinsonian gait (serum levels: 63 ng/ml 87 h after ingestion). Complete resolution of symptoms and signs occurred after 7 days. There is also a single case report of induction of hyponatraemia [103] and another of aripiprazole-induced orthostatic hypotension and cardiac arrhythmia [104]. A very rare side effect is reported in a case report of a woman who developed photo-onycholysis on multiple nails after uptake of olanzapine and subsequent substitution of olanzapine with aripiprazole further exacerbated the problem [105].

## Discussion

Recent reviews suggest that aripiprazole is efficacious in acute mania and in the maintenance treatment of bipolar disorder, with a favourable safety and tolerability profile, with minimal propensity for clinically significant weight gain and metabolic disruption. Aripiprazole should be initiated at 15 mg/day (range 5 to 20 mg/day). If necessary, adjunctive medication should be used in early treatment to manage side effects or assist in management of symptoms such as agitation. When switching to aripiprazole, the therapeutic dose of current treatment should be maintained while adding aripiprazole 15 (5 to 20) mg/day. Only once an effective dose of aripiprazole is reached should previous medication be reduced [106].

A meta-analysis of the two 3-week acute mania RCTs [68,69] suggests that aripiprazole is effective in all subpopulations irrespective of the baseline MADRS score and the presence of rapid cycling, with the exception of remission in patients over 55 years of age [107]. Another meta-analysis of these same RCTs suggested that aripiprazole was superior to placebo in reducing the severity of both mania and agitation in highly agitated patients with bipolar I disorder and showed significant antimanic activity in patients with low levels of agitation without increasing agitation. These findings suggest that aripiprazole's antimanic effect is specific and not limited to control of agitation through sedation [108].

Specifically concerning the effectiveness of aripiprazole against agitation, meta-analytic studies suggest that although non-sedated patients with bipolar I disorder showed significant decreases in PANSS-Excited component scores following treatment with aripiprazole intramuscularly, which is approved for the treatment of agitation associated with schizophrenia or bipolar I disorder (manic or mixed) and this improvement was similar to that seen in patients with schizophrenia, the latter showed significant reductions in PANSS-Excited component scores compared with placebo regardless of baseline level of agitation. In contrast, patients with bipolar I disorder who had higher baseline agitation showed similar improvement on either aripiprazole or placebo while those with lower baseline agitation improved significantly more on aripiprazole [109,110].

The systematic review and meta-analysis of 13 randomised, placebo-controlled trials (involving 3,089 subjects) in acute bipolar mania, which included two aripiprazole studies, suggested a response to treatment (at least 50% reduction in YMRS scores) more than 1.7 times higher in comparison to placebo (relative risk (RR) = 1.74, 95% confidence interval (CI) 1.54 to 1.96). Small but significant increases in extrapyramidal side effects occurred with risperidone and aripiprazole [111]. Another meta-analysis on 15 RCTs and 2,022 patients suggests that aripiprazole is more effective than haloperidol (which in turn is more effective than placebo) at reducing manic symptoms, both as monotherapy and as adjunctive treatment to lithium or valproate and equally effective as olanzapine or risperidone [112]. Another systematic review and meta-analysis from 12 placebo-controlled monotherapy and 6 placebo-controlled adjunctive therapy trials involving a total of 4,304 subjects (including 1,750 placebo-treated subjects) of atypical antipsychotics for acute bipolar mania reports that aripiprazole, olanzapine, quetiapine, risperidone, and ziprasidone all demonstrated significant efficacy in monotherapy without any significant differences in efficacy among antipsychotics. The magnitude of improvement was similar whether the antipsychotic was utilised as monotherapy or adjunctive therapy [113].

Conclusively, the data on the effectiveness of aripiprazole against acute manic/mixed episodes are strong, and so are the data concerning the prophylaxis against these episodes in patients who experienced predominantly manic episodes and whose manic episodes responded to aripiprazole treatment. There are some data suggesting aripiprazole is effective in rapid cycling patients. The data against acute bipolar depression are negative although the research on lower dosages (5 to 10 mg daily) could be warranted since aripiprazole initially seemed to provide positive results in comparison to placebo, which however did not last. There are significant data for the usefulness of aripiprazole as adjunctive therapy to lithium or valproate in refractory bipolar depressive patients. The majority of trials included patients with moderate to severe manic episodes, some of whom also had psychotic symptoms.

With regards to safety, aripiprazole was generally reported to be safe and well tolerated. The adverse effects of aripiprazole were generally mild to moderate and similar to those previously observed in the schizophrenia population treated with aripiprazole. It is important that the adverse effect profile of aripiprazole differs from that of the SGAs since aripiprazole did not show any safety concerns on QT prolongation, hyperprolactinaemia, or weight gain. However, EPS such as akathisia was more frequently reported in aripiprazole-treated than in placebo-treated patients, but at a rate of approximately half that of haloperidol-treated patients; this could still could be treatment limiting in some cases. The hazard risk for tardive dyskinaesia with aripiprazole in the bipolar population is unknown [114,115].

## Competing interests

KNF is a member of the International Consultation Board of Wyeth for desvenlafaxine and has received honoraria for lectures from AstraZeneca, Servier, Janssen-Cilag, Eli Lilly and research grants from AstraZeneca, Janssen-Cilag, Elpen and Pfizer Foundation.

EV has acted as consultant, received grants, or received honoraria for lectures by the following companies: Almirall, AstraZeneca, Bial, Bristol-Myers-Squibb, Eli Lilly, Forrest Research Institute, GlaxoSmithKline, Janssen-Cilag, Jazz Lundbeck, Merck Sharpe Dohme, Novartis, Organon, Pfizer, Sanofi, Servier and UBC.

## Authors' contributions

Both authors independently reviewed the entire literature and contributed equally to the authoring of the paper
